# Bioinformatics analysis and experimental validation of ferroptosis genes in heart failure and atrial fibrillation

**DOI:** 10.3389/fgene.2025.1541342

**Published:** 2025-07-02

**Authors:** Zhi Wang, Chi Yuan, Tao Xu, Weixing Xie, Jiehua Wu, Hegui Wang

**Affiliations:** ^1^ Department of Cardiology, Huangshan Shoukang Hospital, Huangshan, China; ^2^ Department of Cardiology, First Affiliated Hospital of Anhui Medical University, Hefei, China; ^3^ Department of Cardiology, Yijishan Hospital of Wannan Medical College, Wuhu, China

**Keywords:** heart failure, atrial fibrillation, ferroptosis, bioinformatics analysis, biomarker

## Abstract

**Background and objectives:**

Atrial fibrillation (AF) and heart failure (HF) are common cardiovascular diseases associated with significant morbidity and mortality in patients with both conditions. The objective of this research is to enhance our understanding of the shared pathogenesis underlying the two diseases and to identify novel therapeutic targets.

**Materials and methods:**

Differentially expressed genes (DEGs) in heart failure and atrial fibrillation were obtained through the analysis and comparison of transcriptional expression profiles from the Gene Expression Omnibus (GEO) datasets. By integrating these datasets with the known ferroptosis-related genes (FRGs) from GeneCards and PubMed, we identified ferroptosis-related differentially expressed genes (FRDEGs). Functional enrichment and the construction of the PPI network for key genes were conducted. The mRNA-miRNA and mRNA-TF Regulatory Network were constructed via the ChIPBase and TarBase databases. Receiver operating characteristic (ROC) was utilized to screen out the FRDEGs and validate their diagnostic values. Gene expression levels were detected by qPCR in patient serum samples.

**Results:**

By analyzing the transcriptional expression profiles of the GEO datasets, *TFRC*, *CP*, *SAT1*, *STEAP3*, *AKR1C1* and *LPCAT3* were identified as FRDEGs in AF and HF, which were revealed to be involved in iron ion transport, homeostasis, and oxidoreductase activity. Further insights from Gene Set Enrichment Analysis (GSEA) indicated that FRDEGs are primarily enriched in the IL-12 signaling pathway in HF and significantly enriched in the collagen assembly pathway in AF. The diagnostic efficacy of six genes in AF validation sets was good (AUC:*TFRC* 0.940, *CP* 0.920, *SAT1* 1.000, *STEAP3* 0.960, *AKR1C1* 0.900, *LPCAT3* 0.960, as well as in the HF validation set (AUC: *TFRC* 0.842, *CP* 0.879, *SAT1* 0.865, *STEAP3* 0.787, *AKR1C1* 0.812, *LPCAT3* 0.696).Utilizing the GOSemSim package, we conducted a functional similarity analysis on the five hub genes and discovered their significant roles in disease, ranked as follows: *STEAP3*>*TFRC*>*CP*>*SAT1*>*LPCAT3*. qRT-PCR verified the expression differences of *CP*, *STEAP3*, and *LPCAT3*.

**Conclusion:**

Our findings provide a theoretical basis for the clinical diagnosis and treatment of AF and HF. These results provide valuable insights into potential biomarkers for diagnosis and targets for therapeutic intervention in AF and HF.

## 1 Introduction

Atrial fibrillation (AF) and heart failure (HF) are prevalent cardiovascular diseases that frequently co-occur, significantly impacting patient morbidity and mortality worldwide. Krijthe et al. (2013) estimate that there will be 17.9 million cases of AF in Europe by 2060, based on predictions from 2010. Similarly, Ambrosy et al. (2014) believe that HF is a global pandemic affecting an estimated 26 million people worldwide and resulting in over 1 million hospitalizations annually in both the United States and Europe. Research indicates that over half of HF patients develop AF either at onset or during follow-up, leading to higher hospitalization rates, reduced quality of life, and increased mortality (Reddy et al., 2022).

The complex causes of AF and HF remain largely unknown. In patients with both AF and HF, the risks are significantly elevated. HF could predispose individuals to AF due to increased atrial pressure and volume overload, while AF could exacerbate HF by reducing cardiac output and promoting poor ventricular remodeling (Fauchier et al., 2023).

Current treatment strategies for AF and HF primarily consist of pharmacological interventions, including anticoagulants, β-blockers, and antiarrhythmic drugs, alongside non-pharmacological methods such as catheter ablation and device implantation (Kotecha et al., 2014; Chang et al., 2017; Lopes et al., 2018; Marrouche et al., 2018). However, the long-term efficacy and safety of these treatments in HF patients remain uncertain and might lead to various side effects and complications (Roy et al., 2008). These limitations highlight the need for novel therapeutic targets and strategies to improve the management of AF and HF.

Ferroptosis is an iron-dependent process that is morphologically, biochemically, and genetically distinct from apoptosis, necrosis, and autophagy. Its mechanism involves abnormal iron metabolism, lipid peroxidation, and antioxidant system imbalance, ultimately leading to cell death (Dixon et al., 2012). Ferroptosis plays a crucial role in various heart diseases, including atherosclerosis, drug-induced HF, myocardial ischemia-reperfusion injury (IRI), arrhythmia, and diabetic cardiomyopathy (Fang et al., 2023). Several recent studies have confirmed that ferroptosis is a potential therapeutic target for various cardiovascular diseases (CVDs), including cardiomyopathy, myocardial infarction (MI), myocardial IRI, and HF (Bai et al., 2018; Fang et al., 2019; Kitakata et al., 2021).

Ferroptosis Related Genes (FRGs) are involved in a variety of pathological processes, such as oxidative stress, inflammation, and cell death, which are also key features of AF (Heijman et al., 2021) and HF (Zhang et al., 2023). However, the potential pathogenesis and biomarkers of AF and HF have not been fully elucidated, and there are still many related genes to be identified.

Understanding the differential expression of genes associated with ferroptosis in AF and HF could provide new insights into the molecular mechanisms of these conditions and identify potential therapeutic targets.

This study aims to analyze the differential expression of FRGs in AF and HF. By revealing their common biological functions and mechanisms, we can gain insights into the molecular mechanisms of FRGs in AF and HF. In addition, this study seeks to identify potential targets for therapeutic intervention.

## 2 Materials and methods

### 2.1 Data acquisition and preprocessing

We used the GEOquery (Davis and Meltzer, 2007) (Version 2.70.0) package from the GEO database (https://www.ncbi.nlm.nih.gov/geo/) to download gene expression data for the AF-related dataset GSE2240 (Barth et al., 2005) and the HF-related dataset GSE21610 (Schwientek et al., 2010). Both datasets were from *Homo sapiens*. GSE2240 contained 35 samples: 10 from AF patients (designated as group: AF), 5 from healthy controls (designated as group: Control), and 20 from patients in sinus rhythm. For the current analysis, only the AF patient samples and control samples were included (*n* = 15). The data platform used was GPL96. The data platform used for the GSE21610 dataset was GPL570. It contains a total of 68 samples: 30 samples from HF patients (designated as group: HF), eight samples from control subjects (designated as group: Control), and 30 samples from HF patients supported by mechanical circulatory assist devices. For the current analysis, only the HF patient samples and control samples were included (total *n* = 38). The specific data set information is shown in Supplementary Table 1.

The GSE2240 (AF_Dataset) and GSE21610 (HF_Dataset) datasets were standardized by R package limma (Ritchie et al., 2015) (Version 3.58.1), and the annotation probes were standardized and normalized.

The GeneCards (Stelzer et al., 2016) database (https://www.genecards.org/) is an integrative database of human gene information. We used the term “Ferroptosis” as a search keyword and kept only FRGs with “Protein Coding” and “Relevance Score > 2,” and “Ferroptosis” was used as a keyword on the Pubmed website (https://pubmed.ncbi.nlm.nih.gov/) in the published literature on 60 FRG sets (Xiao et al., 2022), with the 119 FRGs received.

### 2.2 Differentially expressed gene analysis

To identify differentially expressed genes (DEGs) between the AF and HF groups, we analyzed the expression profile data from the AF_Dataset and HF_Dataset using the limma package in R. DEGs were defined as genes with |logFC| > 0.5 and *p-*value < 0.05. Genes with logFC > 0.5 and p-value < 0.05 were classified as upregulated DEGs. Genes with logFC < −0.5 and p-value < 0.05 were classified as downregulated DEGs.

To identify the ferroptosis-related differentially expressed genes (FRDEGs), we intersected the DEGs with the curated list of ferroptosis-related genes (FRGs). A Venn diagram was then created to visualize the overlap between these gene sets. The results of the differential analysis were used to create a volcano plot using the R package ggplot2 (Version 3.4.4). A heatmap was generated using the R package pheatmap (Version 1.0.12).

### 2.3 Gene Ontology and pathway enrichment analysis

We performed Gene Ontology (GO) and pathway (KEGG) enrichment analysis on FRDEGs using the clusterProfiler package (Version 4.10.0) in R (Yu et al., 2012). A significance threshold *p-*value of 0.05 and FDR value (adjusted *p-*value) < 0.25 was applied.

### 2.4 Gene Set Enrichment Analysis (GSEA)

In this study, we first sorted the genes from the AF_Dataset and HF_Dataset by logFC. Subsequently, we used the clusterProfiler package to perform Gene Set Enrichment Analysis (GSEA) on all genes related to the differential analysis. The parameters used in the GSEA enrichment analysis were as follows: The seed was 2022, the number of calculations was 5000, and the minimum and maximum number of genes per set was 10 and 500, respectively. Gene sets were obtained from the MSigDB (Liberzon et al., 2015) database (“c2. All. V2022.1. Hs. Symbols. GMT [all Canonical Pathways] (3050)”), focusing on *Homo sapiens*. The screening criteria for significant enrichment were *p-*value < 0.05 and FDR value (adjusted *p-*value) < 0.25.

### 2.5 Protein–protein interaction network and functional similarity analysis

We employed the STRING (Szklarczyk et al., 2019) database to identify interacting proteins for our hub genes. We set the biological species as *Homo sapiens* and used a minimum correlation coefficient greater than 0.400 as the standard for interaction. The resulting protein–protein interaction (PPI) network was visualized using Cytoscape software (Shannon et al., 2003).

The GO semantic similarity of hub genes was calculated using the GOSemSim (Yu et al., 2010) package (Version 2.28.0). The geometric mean of the similarity scores at the biological process (BP), cellular component (CC), and molecular function (MF) levels were used to obtain a final score for each hub gene. Finally, the ggplot package (Version 3.4.4) was used to visualize the functional similarity analysis results. In addition, we utilized the GeneMANIA (Franz et al., 2018) online website to identify genes with functional similarity to our hub genes. We also downloaded the corresponding interaction network from GeneMANIA.

### 2.6 mRNA–TF and mRNA–miRNA interaction network construction

We searched the CHIPBase (Zhou et al., 2017) database (version 3.0) to identify transcription factors (TFS) that bind to our hub genes. Interaction pairs were selected based on the criteria of having a sum of the “number of samples found (upstream)” and “number of samples found (downstream)” greater than 10. The mRNA–TF interaction network was then visualized using Cytoscape software.

To analyze the relationships between hub genes and miRNAs, we searched three databases: TarBase (Vlachos et al., 2015) (http://www.microrna.gr/tarbase), miRDB database (Chen and Wang, 2020) (https://mirdb.org/), and StarBase V3.0 database (Li et al., 2014) (https://starbase.sysu.edu.cn/). The identified mRNA–miRNA regulatory network was then visualized as a network using Cytoscape software.

### 2.7 Differential expression analysis and ROC curve analysis of FRDEGs

To explore the expression patterns of FRDEGs within the AF_Dataset and HF_Dataset, we generated group comparison maps based on their expression levels. In addition, we employed the pROC package in R (Version 1.18.5) to generate ROC curves for the FRDEGs. The Area Under the Curve (AUC) value was calculated for each ROC curve to evaluate the diagnostic potential of FRDEG expression levels in distinguishing between disease states.

### 2.8 Quantitative reverse transcription polymerase chain reaction (qRT-PCR)

Total RNA was extracted from serum samples using TRIzol^®^ (Invitrogen, USA). RNA concentration was measured using a spectrophotometer (BioTek, USA). cDNA synthesis was performed using the Servicebio^®^ RT First-Strand cDNA Synthesis Kit (product number G3330) according to the manufacturer’s instructions. Briefly, reverse transcription was carried out at 42°C for 60 min, followed by enzyme inactivation at 70°C for 5 min. qRT-PCR was performed on the Light Cycler^®^ 4800 system (Roche Diagnostics) using a specific set of primers designed to amplify the genes of interest. The thermal cycling conditions used were as follows: 95°C for 15 s, followed by 60°C for 60 s (for a total of 30 cycles). β-actin was used as an endogenous control for normalization. The relative quantification was calculated using the ΔΔCt method. Specific primer sequences are listed in Supplementary Table 2.

### 2.9 Technology roadmap

**FIGURE 1:**
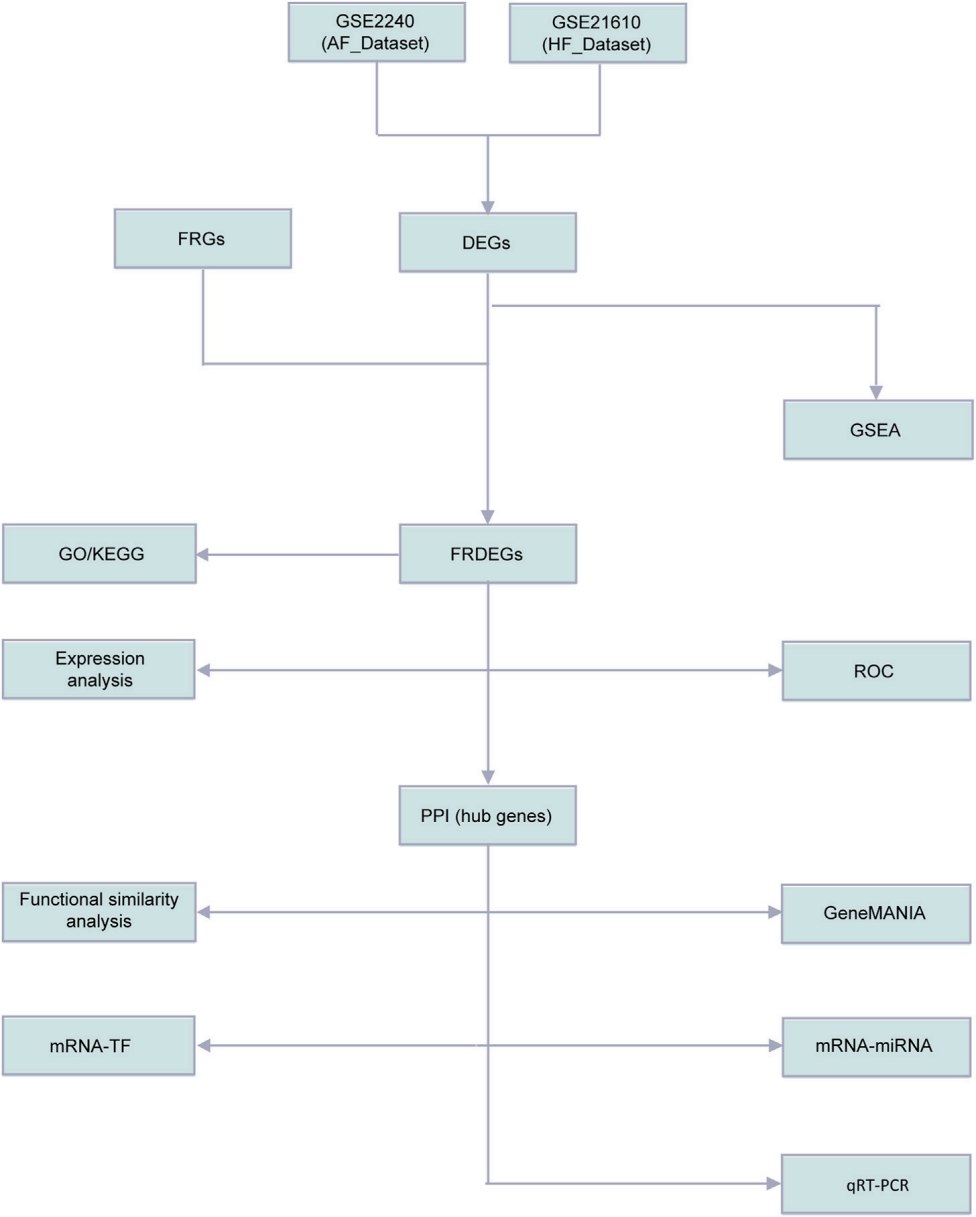
Flow chart of methodologies applied in the study. AF, Atrial fibrillation; HF, Heart failure; FRGs, Ferroptosis Related Genes; FRDEGs, Ferroptosis Related Differentially Expressed Genes; DEGs, Differentially expressed genes; GSEA, Gene Set Enrichment Analysis; GO, Gene Ontology; KEGG, Kyoto Encyclopedia of Genes and Genomes; ssGSEA, single-sample gene-set enrichment Analysis; ROC, Receiver Operating Characteristic Curve; PPI, Protein-protein interaction network; TF, Transcription factors.

**FIGURE 2:**
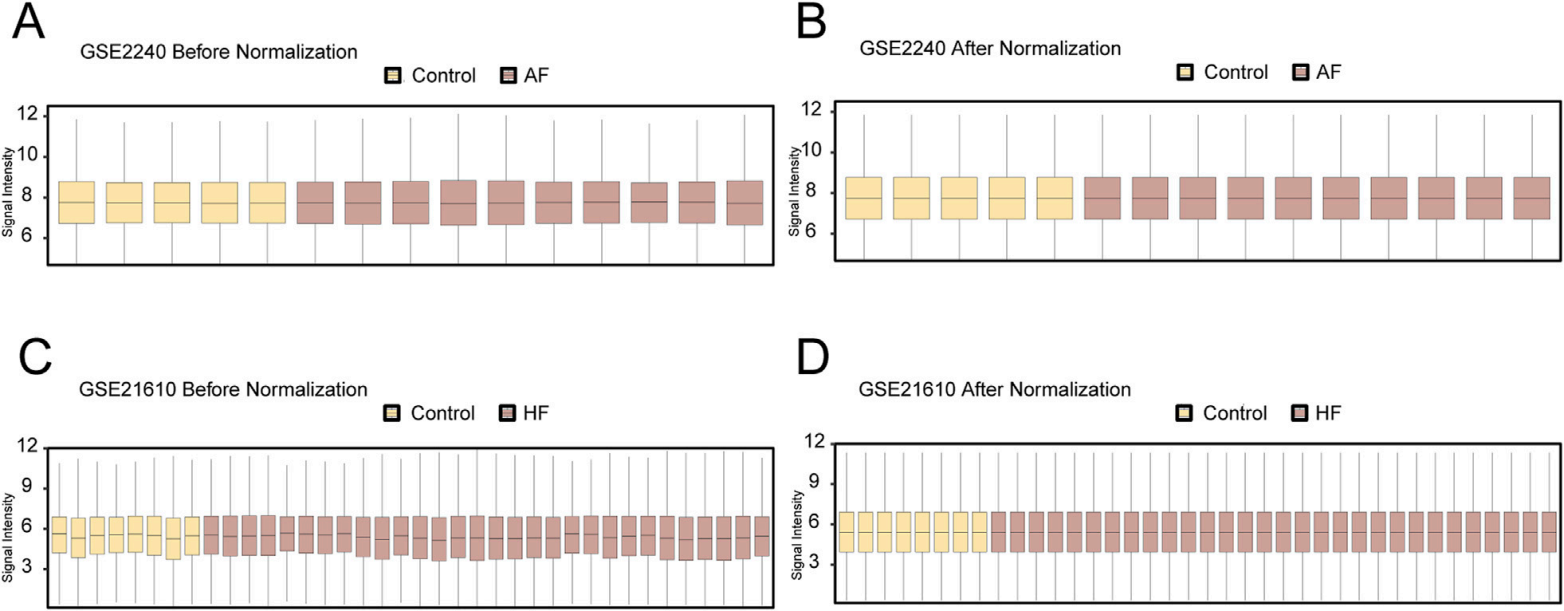
Dataset correction. **(A)** Boxplot of the GSE2240 dataset before correction. **(B)** Boxplot plot of the corrected GSE2240 dataset. **(C)** Boxplot plot of the GSE21610 dataset before correction. **(D)** Boxplot plot of the corrected GSE21610 dataset. Yellow is the Control group, and brown is the AF and HF groups.

The workflow of this study was presented in Figure 1. To remove batch effects in the dataset, we first used the limma R package to perform standardized correction on the datasets GSE2240 (AF_Dataset) and GSE21610 (HF_Dataset) and compared the datasets before and after correction through the distribution boxplot (Figures 2A–D). The results of the distribution boxplot showed that the batch effects of the dataset were removed.

### 2.10 Statistical analysis

The data processing and analysis for this paper were conducted using R software (Version 4.2.2). To compare continuous variables between two groups, the statistical significance of normally distributed variables was estimated using the independent samples *t*-test. The Mann–Whitney U test (Wilcoxon rank sum test) was used to analyze the differences between variables that were not normally distributed. For comparisons involving more than three groups, the Kruskal–Wallis test was employed. The chi-square test or Fisher’s exact test was used to compare and analyze statistical significance between the two groups of categorical variables. Spearman correlation analysis was used to calculate the correlation coefficient between different molecules. During the processing of qRT-PCR data, one-way ANOVA was performed to compare the groups. All statistical *p-*values are two-sided unless otherwise specified. A *p-*value of less than 0.05 was considered to indicate statistical significance.

## 3 Results

### 3.1 Analysis of differentially expressed genes

**FIGURE 3:**
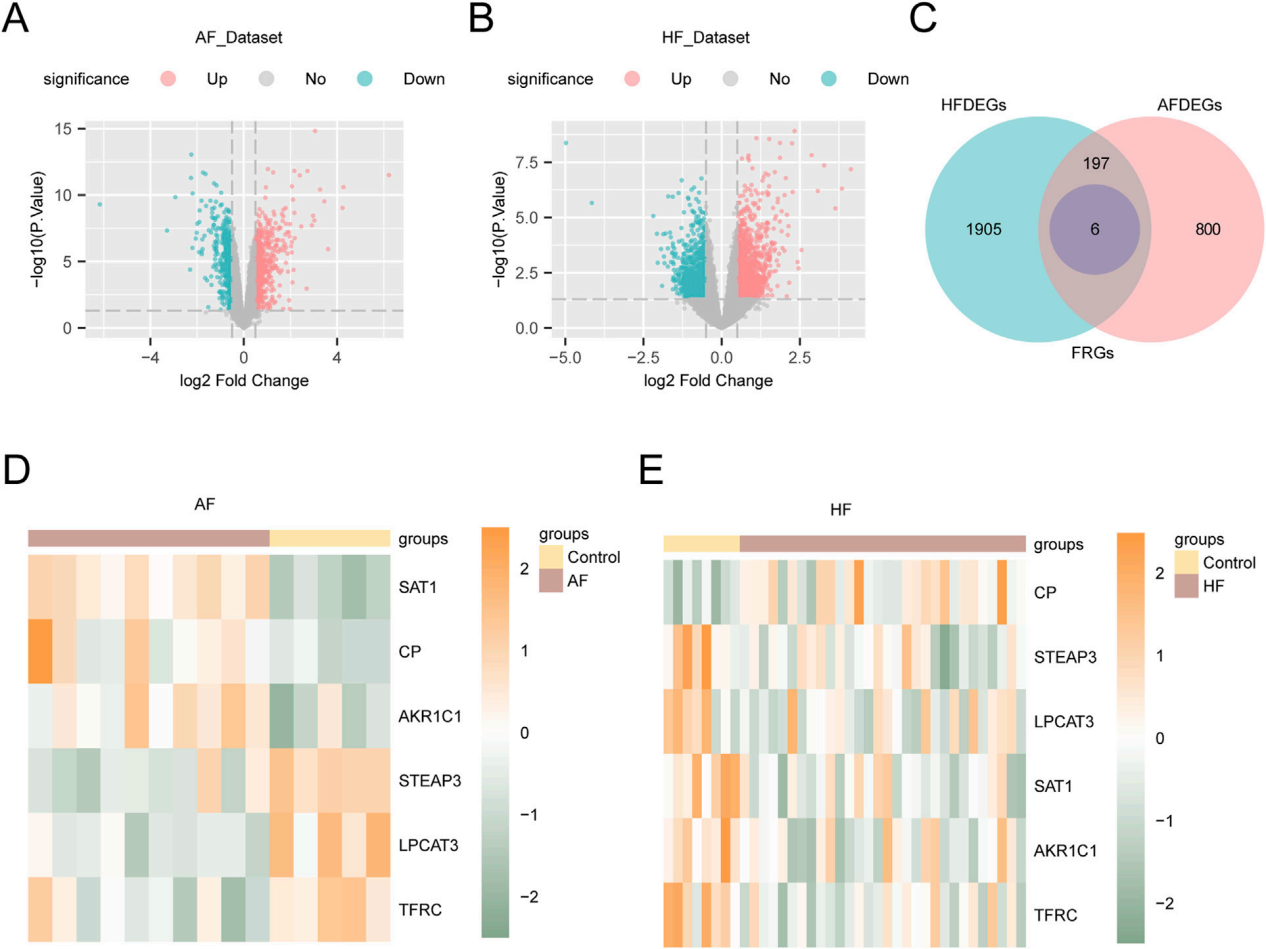
Analysis of differentially expressed genes. **(A)** Volcano plot of the results of differential analysis between AF and Control groups in the AF_Dataset. **(B)** Volcano plot of difference analysis results between HF and Control groups in HF_Dataset. **(C)** The Venn diagram showing the intersection between the FRGs and the DEGs in AF_Dataset and HF_Dataset. **(D)** Differential expression heatmap of FRDEGs in AF_Dataset. **(E)** Heat map of differential expression of FRDEGs in HF_Dataset. Yellow is the Control group, and brown is the AF and HF groups. Green represents low expression and orange represents high expression in the heat map.

To identify DEGs between AF and HF groups, we analyzed the AF_Dataset and HF_Dataset using the limma R package. The analysis revealed that the AF_Dataset contained 1003 genes meeting the criteria of |logFC| > 0.5 and p-value < 0.05, including 555 upregulated genes (logFC > 0.5) and 448 downregulated genes (logFC < −0.5). A volcano plot was generated based on the differential analysis results of this dataset (Figure 3A). The HF_Dataset included a total of 2108 genes that met the thresholds of |logFC| > 0.5 and p-value < 0.05. Among these, 1139 genes were upregulated (logFC > 0.5) and 969 were downregulated (logFC < −0.5). The corresponding volcano plot is shown in Figure 3B. The full results of the DGE analysis are presented in Supplementary Tables 3 and 4.

To obtain the FRDEGs, we analyzed the AF_Dataset and HF_Dataset. Next, we intersected all DEGs with FRGs that met the criteria (|logFC| > 0.5 and p-value < 0.05). Then, a Venn diagram was created to illustrate the results (Figure 3C). In total, we identified six FRDEGs: *TFRC, CP, SAT1, STEAP3, AKR1C1,* and *LPCAT3*. According to the intersection results, the expression differences of FRDEGs between different sample groups in the dataset AF_Dataset (Figure 3D) and HF_Dataset (Figure 3E) were analyzed, and the pheatmap package in R was used to visualize the results.

### 3.2 Functional enrichment analysis and pathway enrichment analysis

**FIGURE 4:**
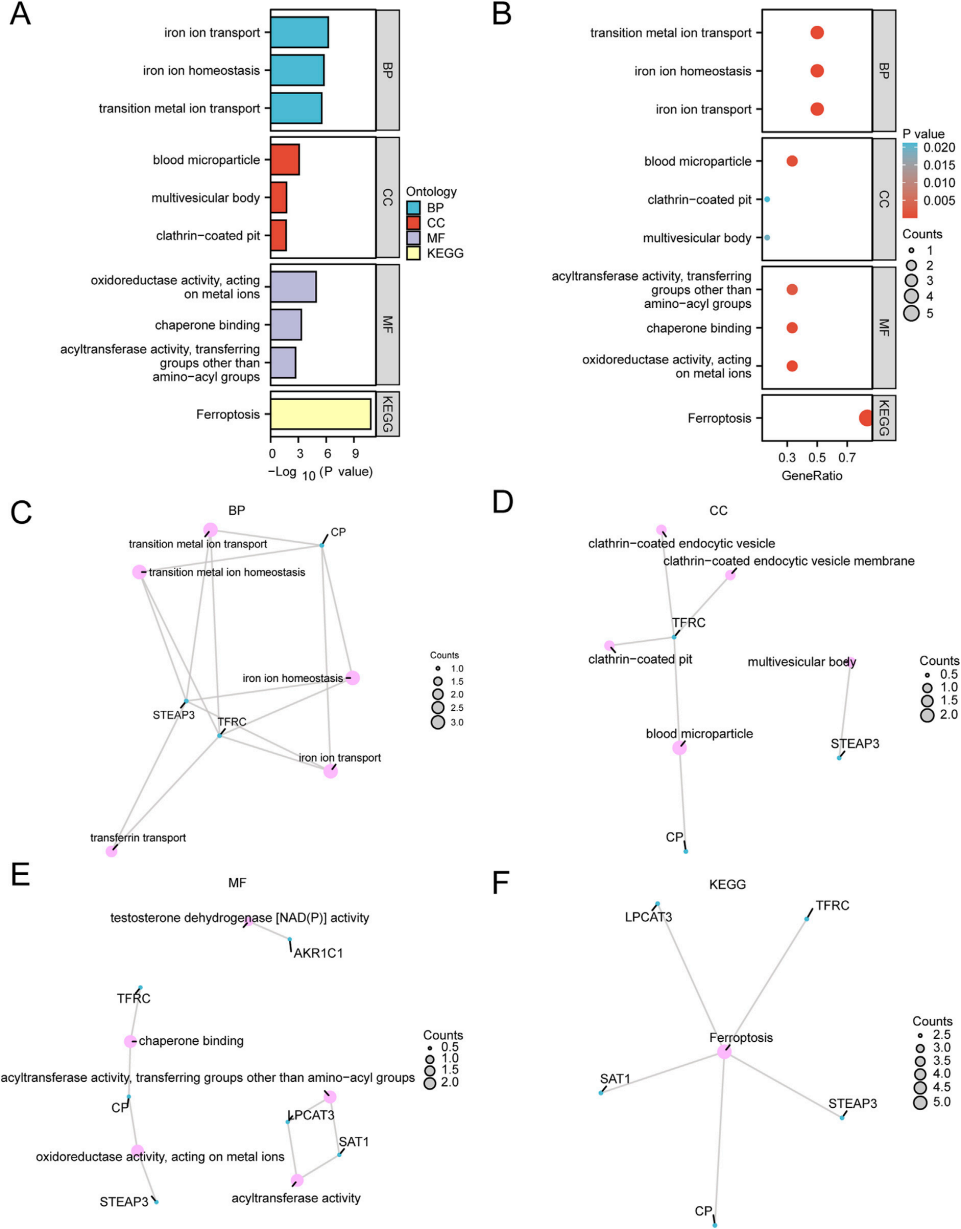
Functional enrichment analysis (GO) and pathway enrichment (KEGG) analysis. **(A,B)** Gene Ontology (GO) and pathway (KEGG) enrichment analysis results of differentially expressed genes related to ferroptosis bar graph **(A)** and bubble plot **(B)** show the biological process (BP), cell component (CC) and biological pathway (KEGG). GO terms and KEGG terms are shown on the ordinate. **(C–F)** Gene Ontology (GO) and pathway (KEGG) enrichment analysis results of FRDEGs: BP **(C)**, CC **(D)**, MF **(E)**, and KEGG **(F)**. Pink dots represent specific pathways, and blue dots represent specific genes. GO, Gene Ontology; BP, biological process; CC, cellular component; MF, molecular function; KEGG, Kyoto Encyclopedia of Genes and Genomes. In the bubble diagram, the size of the bubble represents the number of genes, and the color of the bubble represents the size of the *p-*value. The redder the color, the smaller the *p-*value, and the bluer the color, the larger the *p-*value. The screening criteria for Gene Ontology (GO) and pathway (KEGG) enrichment analysis were *p-*value < 0.05 and FDR value (*p-*value) < 0.25.

To further explore the BP, CC, MF, and biological pathways (KEGG) associated with the six FRDEGs (*TFRC, CP, SAT1, STEAP3, AKR1C1,* and *LPCAT3*)*,* we conducted enrichment analyses. Detailed results are presented in Supplementary Table 5. The results indicated that these six FRDEGs were primarily enriched in the following categories: biological processes such as iron ion transport, iron ion homeostasis, and transition metal ion transport; cellular components, including blood microparticles, multivesicular bodies, and clathrin-coated pits; and molecular functions, such as FRAGE receptor binding, Toll-like receptor binding, and calcium-dependent protein binding. In addition, KEGG enrichment analysis revealed significant enrichment of these FRDEGs in oxidoreductase activity, metal ion interaction, and chaperone binding. The results of the GO and KEGG enrichment analysis are visualized in bar plots (Figure 4A) and bubble plots (Figure 4B). We also created networks for BP (Figure 4C), MF (Figure 4D), CC (Figure 4E), and KEGG pathways (Figure 4F) based on the enrichment analysis results. In these plots, lines represent corresponding molecules and their annotations, while larger nodes indicate a higher number of associated molecules.

### 3.3 Gene Set Enrichment Analysis

**FIGURE 5:**
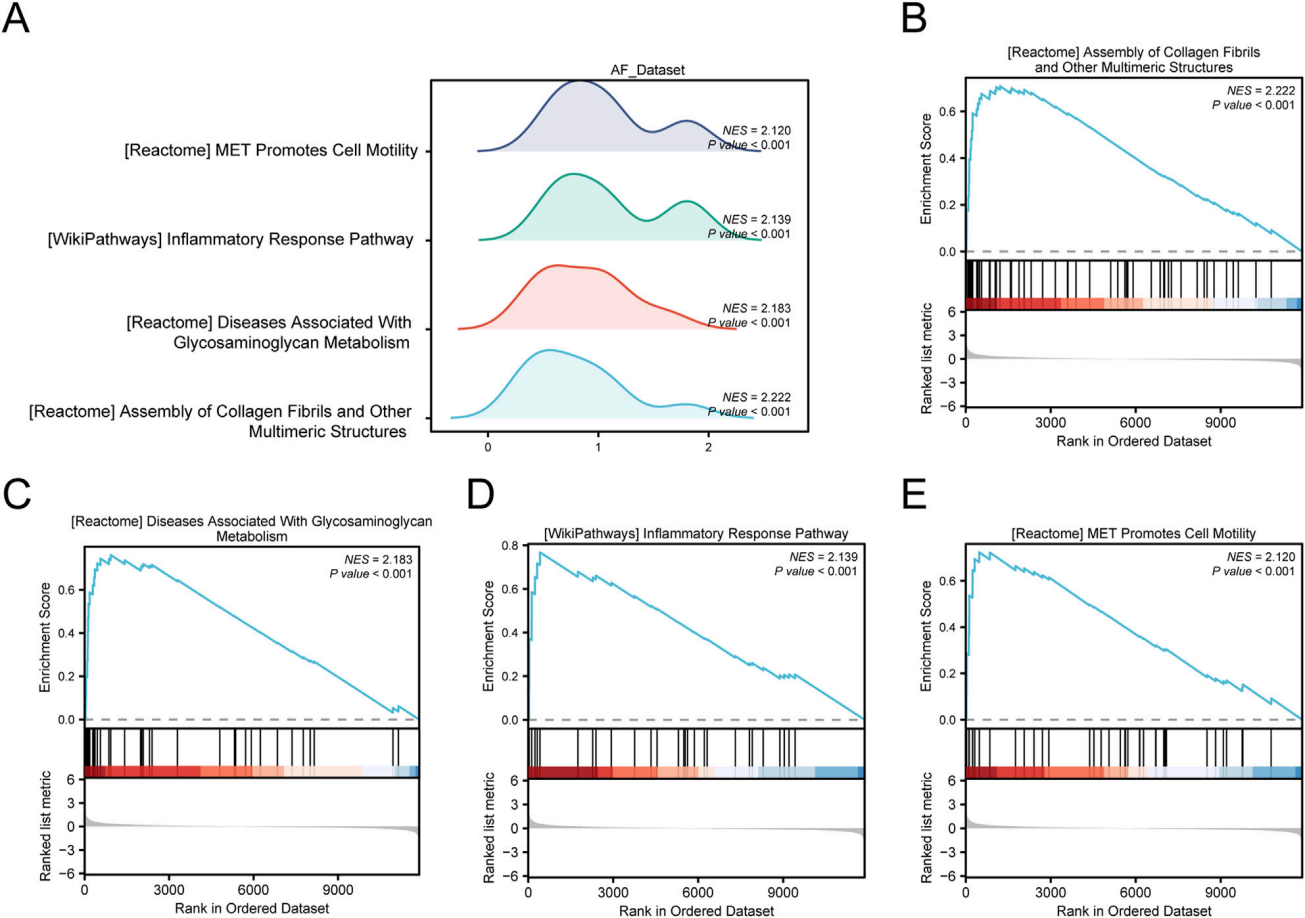
GSEA of the AF dataset. **(A)** Mountain map of four main biological functions by GSEA enrichment analysis of the AF Dataset. B–E. Genes in the AF Dataset were significantly enriched in collagen fibrils **(B)**, glycosaminoglycans metabolism **(C)**, inflammatory response pathway **(D)**, and met pathway **(E)**. The screening criteria for GSEA were *p-*value < 0.05 and FDR value (*p-*value) < 0.25.

To determine the effect of all gene expression levels in the AF_Dataset on disease, GSEA was used to study the relationship between the expression of all genes in the AF Dataset and the biological processes involved, the cellular components affected, and the molecular functions played (Figure 5A). Detailed results are presented in Supplementary Table 6. The results showed that all genes in the AF Dataset were significantly enriched in collagen fibrils (Figure 5B), glycosaminoglycans metabolism (Figure 5C), inflammatory response pathway (Figure 5D), met pathway (Figure 5E), and other biologically related functions and signaling pathways.

**FIGURE 6:**
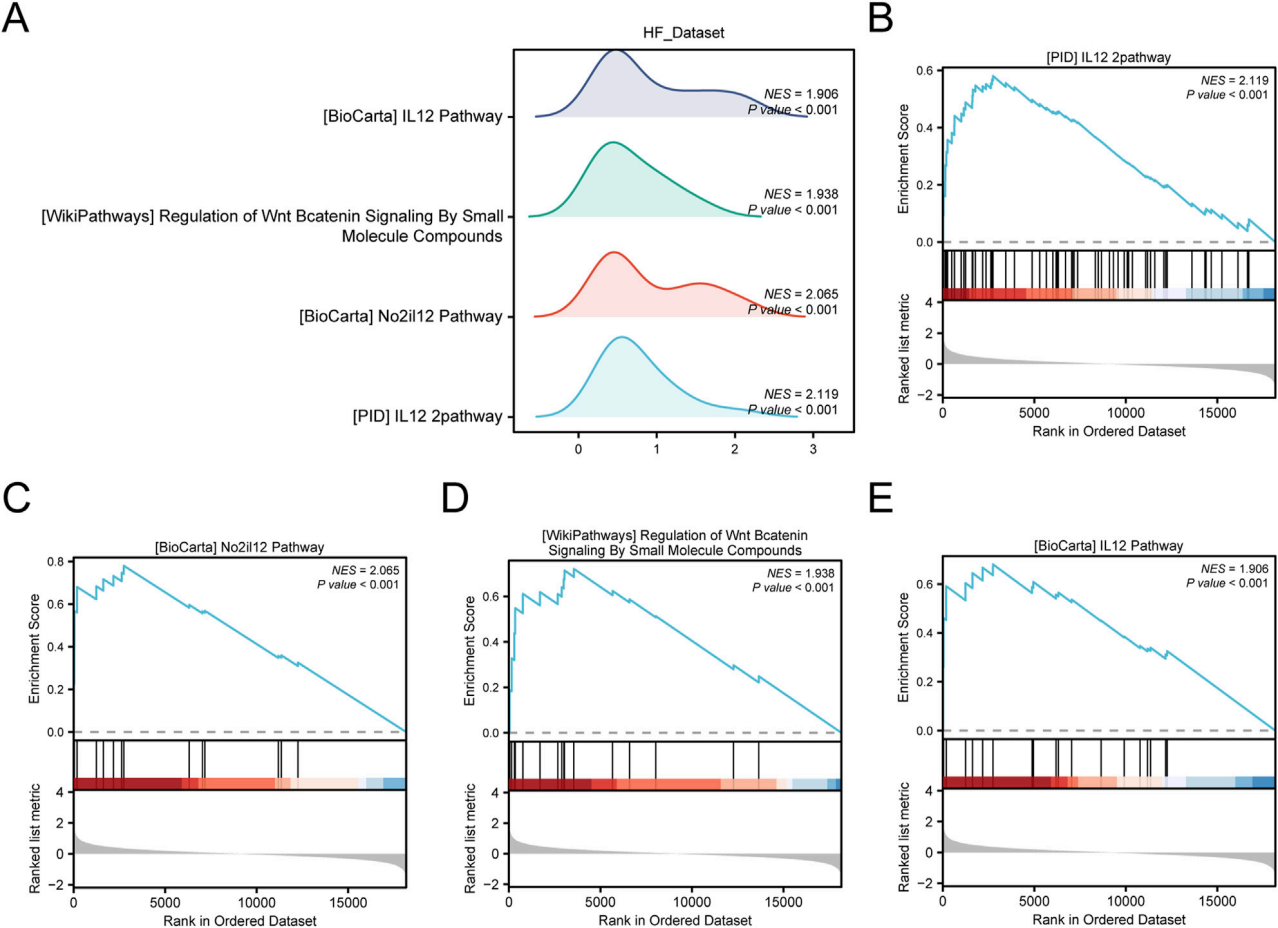
GSEA enrichment analysis of HF_Dataset. **(A)** Mountain map of four main biological functions in GSEA enrichment analysis of HF_Dataset. B–E. Genes in HF_Dataset were significantly enriched in PID_IL12_2 pathway **(B)**, BIOCARTA_NO2IL12 pathway **(C)**, Wnt/β-catenin pathway **(D)**, and IL12 pathway **(E)** GSEA, Gene Set Enrichment Analysis. The screening criteria for GSEA were *p-*value < 0.05 and FDR value (*p-*value) < 0.25.

In addition, we used GSEA to study the relationship between the expression of all genes in the HF_Dataset and the biological processes, cellular components, and their molecular functions (Figure 6A). Detailed results are presented in Supplementary Table 7. The results showed that all genes in the HF_Dataset were significantly enriched in the IL12 pathway (Figure 6B), NO2IL12 pathway (Figure 6C), Wnt/β-catenin pathway (Figure 6D), BIOCARTA_IL12 pathway (Figure 6E), and other biologically related functions and signaling pathways.

### 3.4 PPI interaction network and functional similarity analysis

**FIGURE 7:**
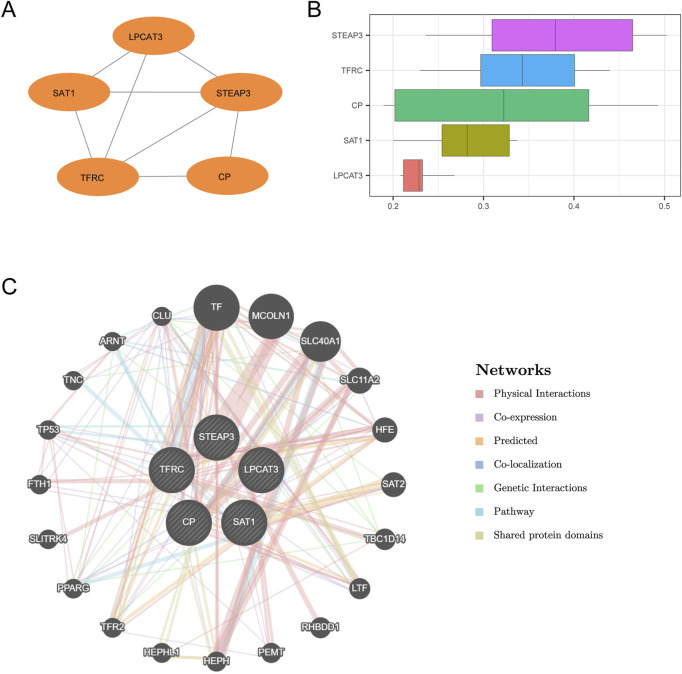
PPI interaction network and functional similarity analysis. **(A)** PPI network of hub genes calculated by the STRING database. **(B)** Box plots of functional similarity of hub genes. **(C)** GeneMANIA website predicts the interaction network of hub genes with similar functions. The circles show the hub genes and the genes with similar functions, and the colors corresponding to the lines represent the interconnected functions.

We conducted a PPI analysis (PPI network, with a low required interaction score set at medium confidence (0.400)) on six FRDEGs (*TFRC, CP, SAT1, STEAP3, AKR1C1, LPCAT3*) utilizing the STRING database. After filtering to retain only those genes that demonstrated connections with other nodes and designated them as hub genes for subsequent analysis, we constructed a PPI network comprising five hub genes (*TFRC, CP, SAT1, STEAP3,* and *LPCAT3*) and visualized this network using Cytoscape software (Figure 7A).

Afterward, we performed functional similarity analysis of the five hub genes utilizing the GOSemSim package, and the results are presented by boxplot (Figure 7B). The results showed that the genes played an important role in the disease, in the order of *STEAP3 > TFRC > CP > SAT1 > LPCAT3*. Finally, the interaction network of five hub genes and their functionally similar genes was constructed by GeneMANIA website prediction (Figure 7C). The lines with different colors represent the co-expression, shared protein domains, co-localization, predicted, pathway, and other information.

### 3.5 Construction of mRNA–TF and mRNA–miRNA interaction network

**FIGURE 8:**
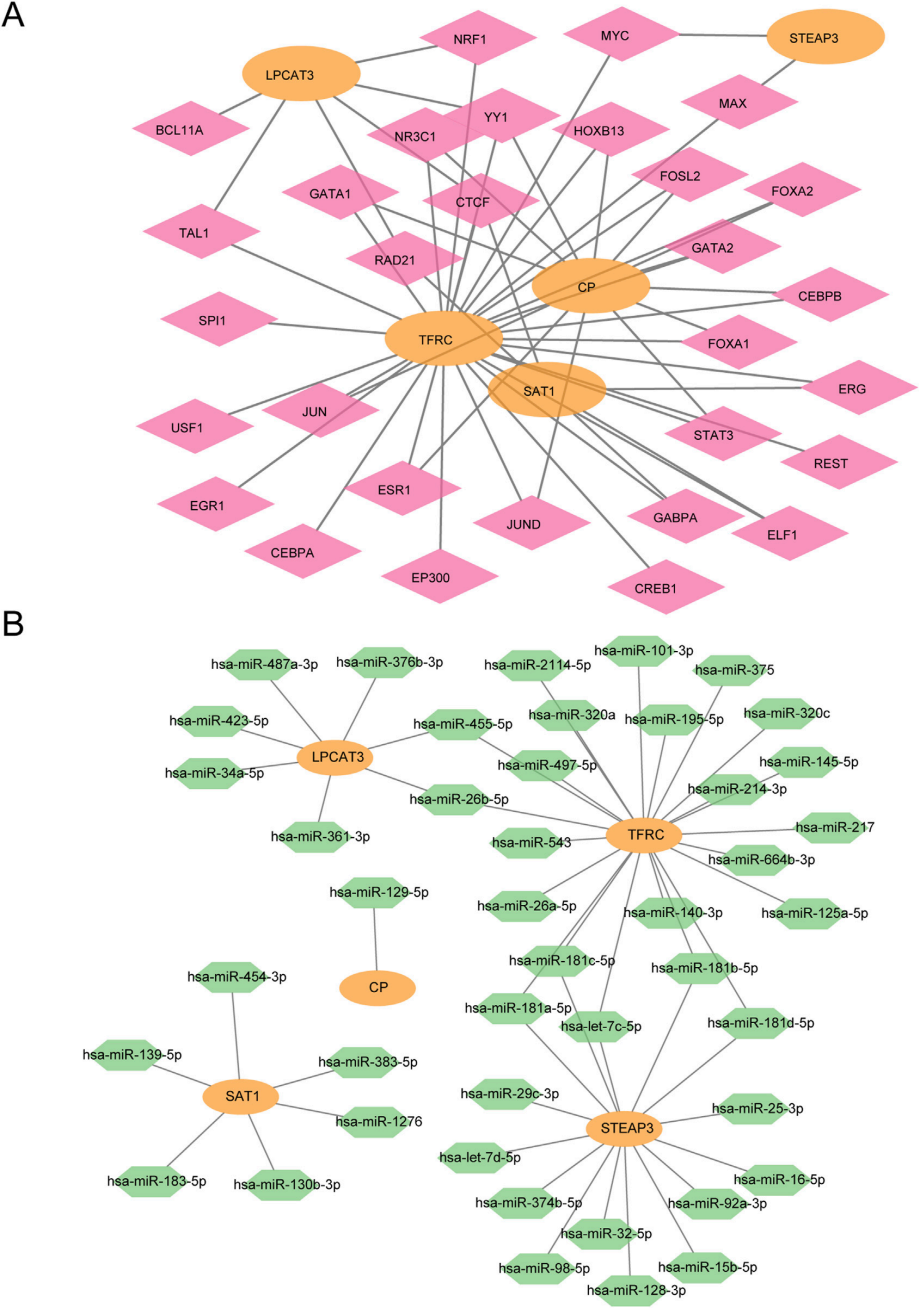
mRNA–TF and mRNA–miRNA interaction network. **(A)** mRNA–TF regulatory network of hub genes. **(B)** mRNA–miRNA regulatory network of hub genes. Orange represents mRNA, pink represents TF, and green represents miRNA.

First, we retrieved the transcription factors (TFs) that bind to the hub genes from the ChIPBase database to construct an mRNA–TF regulatory network. This network was then visualized using Cytoscape software (Figure 8A). Among them, there were 5 hub genes and 30 transcription factors (TFS).

Subsequently, the miRNAs related to the hub genes were obtained through the TarBase database (StarBase database/miRDB database), The mRNA–miRNA regulatory network was constructed and visualized by Cytoscape software (Figure 8B). There were 5 hub genes and 44 miRNAs.

### 3.6 Differential expression analysis and ROC analysis of FRDEGs

**FIGURE 9:**
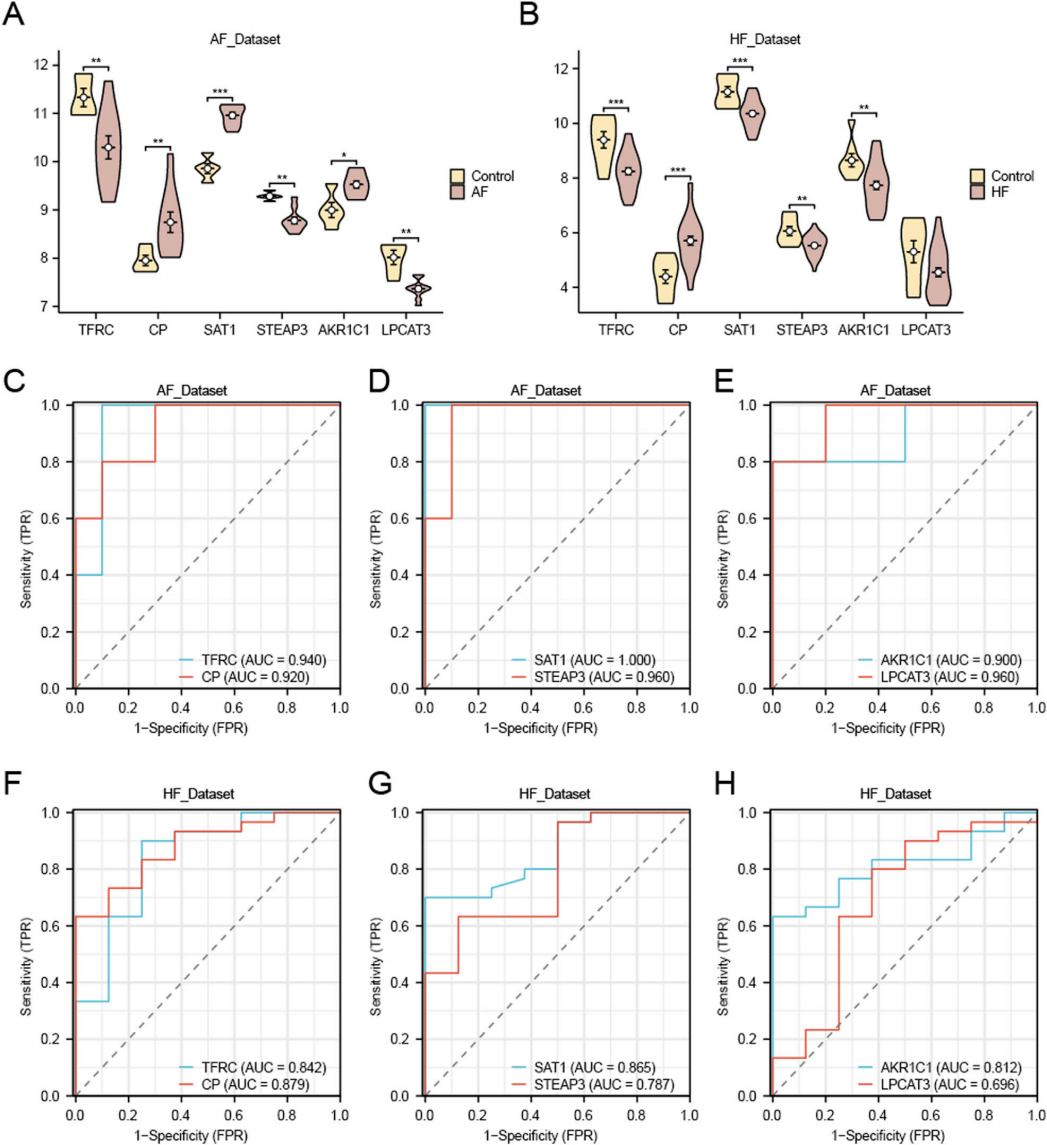
Differential expression analysis and ROC analysis of FRDEGs. **(A)** Group comparison plot of FRDEGs in AF and Control groups in the AF_Dataset dataset. **(C–E)** ROC curves of FRDEGs: *TFRC* and *CP* **(C)**, *SAT1* and *STEAP3* **(D)**, *AKR1C1* and *LPCAT3* **(E)** between different groups of AF_Dataset (AF/Control). **(B)** Group comparison plot of FRDEGs in HF and Control groups in HF_Dataset. F–H. ROC curves of FRDEGs: *TFRC* and *CP* **(F)**, *SAT1* and *STEAP3* **(G)**, and *AKR1C1* and *LPCAT3* **(H)** between different groups of HF_Dataset (HF/Control). The symbol * is equivalent to p-value < 0.05, which is statistically significant. The symbol ** is equivalent to p-value < 0.01, which is highly statistically significant; The symbol *** is equivalent to *p* < 0.001 and highly statistically significant. The closer the AUC in the ROC curve to 1, the better the diagnostic effect. When AUC was between 0.5 and 0.7, the accuracy was low. When AUC was 0.7–0.9, it had a certain accuracy. AUC > 0.9 had high accuracy. Group comparison plots are yellow for the Control group and brown for the AF and HF groups.

We utilized the Wilcoxon rank sum test to analyze the six FRDEGs (*TFRC*, *CP*, *SAT1*, *STEAP3*, *AKR1C1*, and *LPCAT3*) in the AF group and control group of the AF_Dataset (Figure 9A). The results showed that in the AF_Dataset, the expression of six FRDEGs was significantly different between the AF group and the Control group (P < 0.05). Subsequently, the ROC curve was drawn based on the expression of these six FRDEGs in the AF_Dataset (Figures 9C–E). As shown in the figure, the expression of *TFRC* (AUC = 0.940, Figure 9C), *CP* (AUC = 0.920, Figure 9C), *SAT1* (AUC = 1.000, Figure 9D), *STEAP3* (AUC = 0.960, Figure 9D), *AKR1C1* (AUC = 0.900, Figure 9E), and *LPCAT3* (AUC = 0.960, Figure 9E) all had high accuracy in the diagnosis of the disease.

In addition, we used the same method to analyze the six FRDEGs (*TFRC*, *CP*, *SAT1*, *STEAP3*, *AKR1C1*, *LPCAT3*) in the HF group and Control group of the HF_Dataset (Figure 9B). The five FRDEGs had statistically significant differences in expression between the HF group and the Control group (*p* < 0.05). Subsequently, we drew the ROC curve based on the expression of these six FRDEGs in the HF_Dataset (Figures 9F–H). As shown in the figure, the expression of *TFRC* (AUC = 0.842, Figure 9F), *CP* (AUC = 0.879, Figure 9F), *SAT1* (AUC = 0.865, Figure 9G), *STEAP3* (AUC = 0.787, Figure 9G), and *AKR1C1* (AUC = 0.812, Figure 9H) had certain accuracy in the diagnosis of the disease. *LPCAT3* expression demonstrated low diagnostic accuracy for heart failure (AUC = 0.696; Figure 9H).

### 3.7 Quantitative reverse transcription polymerase chain reaction (qRT-PCR)

To evaluate the relationship between five hub genes (*TFRC, CP, SAT1, STEAP3, LPCAT3*) and HF and AF, we collected serum samples from 27 patients (9 healthy, 9 with HF, and 9 with AF) at Huangshan Shoukang Hospital in Anhui Province, China. The case information of all the samples is listed in Supplementary Table 8.

**FIGURE 10:**
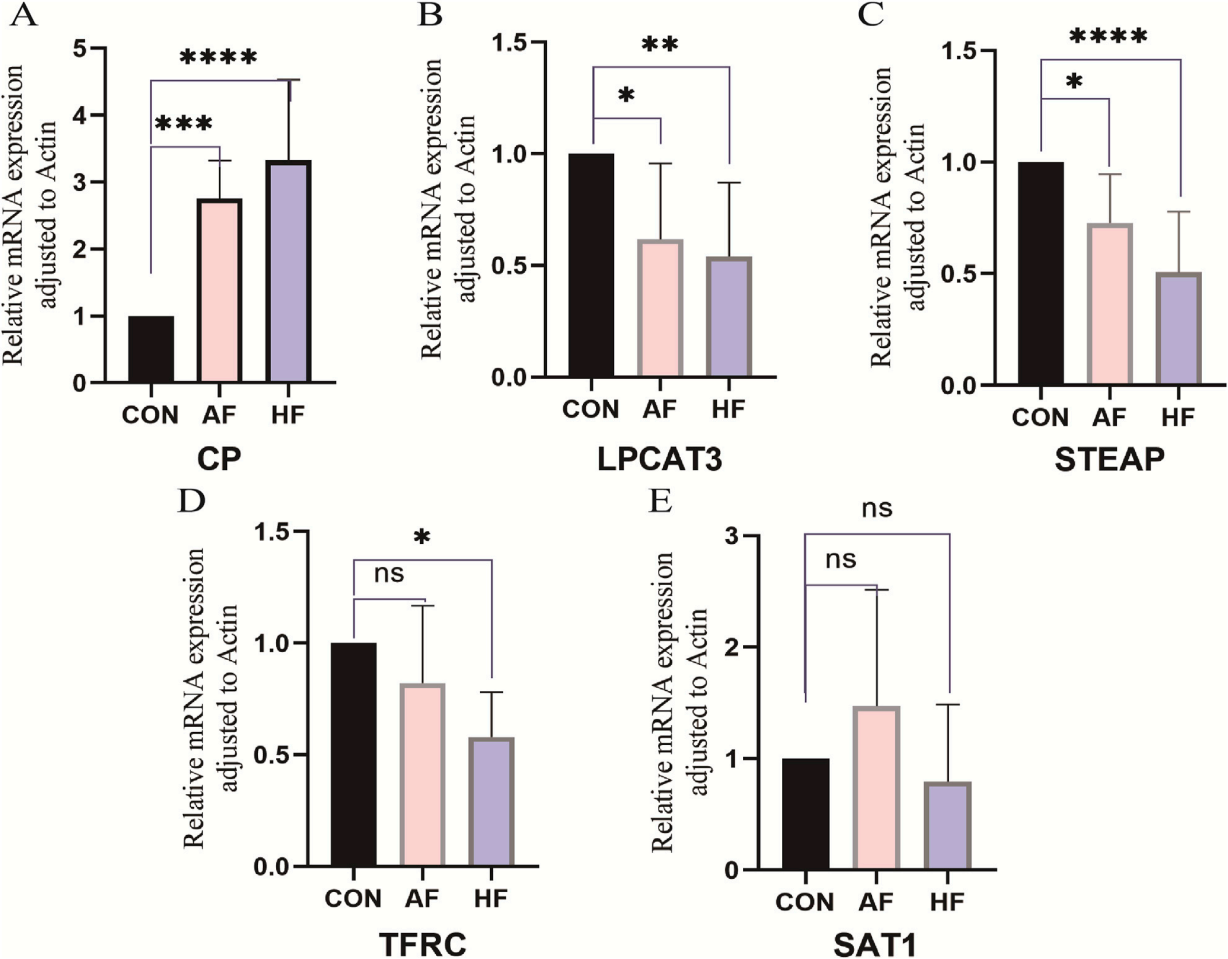
Relative mRNA expression of FRDEGs. Serum samples from patients in HF, AF, and control (CON) groups were analyzed to verify the expression of five hub genes (*TFRC, CP, SAT1, STEAP3, LPCAT3*) using quantitative real-time reverse transcription PCR (qRT-PCR). Data are presented as mean ± standard deviation (SD) with a sample size of *n* = 9. Statistical significance is indicated by the symbol *, representing *p-*value < 0.05; ** denotes a highly significant result, corresponding to *p-*value < 0.01; and ns indicates no statistical significance (*p-*value ≥ 0.05).

We subsequently performed a qRT-PCR experiment to analyze the expression levels of the five hub genes in the serum samples. Figure 10 illustrates that compared with the CON group, *LPCAT3* (Figure 10B), *STEAP3* (Figure 10C) and *TFRC* (Figure 10D) were significantly downregulated in the plasma samples of HF patients (*p-*value < 0.05), while *CP* (Figure 10A) showed a significant increase (*p-*value < 0.05). These findings align with the results of the bioinformatics analysis. The expression of *SAT1* (Figure 10E) did not differ significantly between HF and CON. In AF patients, *LPCAT3* (Figure 10B) and *STEAP3* (Figure 10C) were significantly downregulated in plasma samples compared with the CON group (p-value < 0.05). Conversely, *CP* (Figure 10A) was significantly increased (*p*-value < 0.05), consistent with the bioinformatics analysis. No significant differences were observed in the expression of *TFRC* (Figure 10D) and *SAT1* (Figure 10E) between AF and CON.

## 4 Discussion

HF and AF are complex biological processes with multiple contributing factors and stages. Recent advancements in biomarker discovery have significantly improved early diagnosis, research on pathological mechanisms, and drug target identification for these conditions (Koniari et al., 2021). Ferroptosis, an iron-dependent regulated cell death pathway, is now recognized as a critical driver in the pathogenesis and progression of multiple CVDs. The study demonstrated that development of persistent AF may be prevented by intervention with exosomal miRNAs to reduce oxidative stress injury and ferroptosis (Liu et al., 2022). Iron deficiency or overload perturbs cardiomyocyte iron homeostasis, thereby contributing to HF (Fang et al., 2023). MiR-375-3p Promotes Cardiac Fibrosis by Regulating the Ferroptosis Mediated by GPX4 (Zhuang et al., 2022). However, existing markers provide a limited understanding of the underlying biological and genetic mechanisms of AF and HF, highlighting the need for further investigation.

In this study, we employed various statistical approaches including differential expression analysis, functional enrichment analysis, PPI network construction, interaction network analysis, and qRT-PCR. This comprehensive approach offers deeper insights into the role of FRG compared with previous studies (Ibrahim and Januzzi, 2018; Oikonomou et al., 2019). Notably, recent studies have indicated the critical role of ferroptosis in cardiomyopathy (He et al., 2021; Ta et al., 2022; Wang et al., 2022). Our analysis of gene expression data from AF and HF datasets revealed significant differences in the expression of FRGs. Fang et al. (2024) reviewed the specific role of ferroptosis in AF and HF, emphasizing its involvement in iron regulation, metabolic mechanisms, and lipid peroxidation.

In our analysis, we identified five FRDEGs: *TFRC, CP, SAT1, STEAP3,* and *LPCAT3*. Their dysregulation suggests that iron metabolism disturbances may contribute to the pathogenesis of these conditions.

*TFRC* (Transferrin receptor C) is a transmembrane glycoprotein expressed on the cell membrane that mediates cellular iron uptake. While previous studies have linked *TFRC* in cardiomyocytes to HF progression through macrophage infiltration (Pan et al., 2023), our study showed significantly lower *TFRC* expression in both AF and HF datasets. However, qRT-PCR results revealed a significant reduction only in the HF group. As a potential therapeutic target, the role of *TFRC* in the pathogenesis of HF deserves further attention.

*CP* (Ceruloplasmin) is an acute-phase reactant that is synthesized and secreted by the liver as well as monocytes/macrophages. It participates in both iron and copper metabolism (Fox et al., 2000; Pan et al., 2023). Lazar-Poloczek et al. (2021) identified a correlation between high *CP* and increased mortality in HF patients (Hammadah et al., 2014). In patients with AF, the *CP* gene promoter was strongly associated with increased levels of plasma ceruloplasmin and increased AF risk (Adamsson Eryd et al., 2014). This shows that higher *CP* concentrations were associated with increased AF risk (Arenas de Larriva et al., 2017). Our study revealed that *CP* levels were significantly higher in HF and AF patients, suggesting its potential involvement in the progression of these diseases. Thus, *CP* could be a new therapeutic target for patients with HF and AF.

*LPCAT3* (Lysophosphatidylcholine acyltransferase 3) plays a role in promoting ferroptosis. The lack of *LPCAT3* leads to a marked reduction in membrane arachidonate levels during ferroptosis (Lin et al., 2024). Unlike previous studies (Gawargi and Mishra, 2024), in our study, we found that *LPCAT3* levels were significantly reduced in both HF and AF. However, its accuracy in diagnosing HF (AUC = 0.696) is relatively low, potentially diminishing its predictive utility.

*STEAP3* (six-transmembrane epithelial antigen of the prostate 3) is a member of the STEAP family and is essential for iron and copper uptake. *STEAP3* mRNA is highly expressed in the liver, bone marrow, placenta, skeletal muscle, and heart (Ohgami et al., 2005). Our study revealed a significant reduction of *STEAP3* in both HF and AF, corroborating the results of earlier studies linking it to the negative regulation of pathological cardiac hypertrophy (Li et al., 2020). This suggests *STEAP3* may become a new therapeutic target for patients with coexisting HF and AF.

*SAT1* (spermidine/spermine N1-acetyltransferase 1) is an important regulator in polyamine metabolism. *SAT1* depletion also inhibits p53-induced ferroptosis (Ou et al., 2016). Although our research indicated high diagnostic accuracy of *SAT1* in both conditions, qRT-PCR results did not show a significant difference in its expression levels. Further studies are required to elucidate the roles of *SAT1* in AF and HF development.

Functional enrichment analysis indicated that these FRDEGs are involved in key biological processes, such as iron ion transport and homeostasis, as well as in molecular functions, such as receptor and protein binding. GSEA enrichment analysis further supported their involvement in collagen assembly, glycosaminoglycan metabolism, and inflammatory responses, processes known to play roles in structural and functional remodeling of the heart in AF and HF (Liu et al., 2023). Notably, *STEAP3* emerged as a critical node within the PPI network, indicating its central role in protein interactions relevant to AF and HF. The ROC curve demonstrated its diagnostic value for both conditions, further validated by the subsequent PCR assay. In addition, we identified several key TFs and miRNAs that interact with hub genes. For instance, the mRNA–TF network revealed interactions involving TFs such as *Hobx13*, which is known to regulate cardiomyocyte maturation and proliferation (Liao et al., 2010). Similarly, the mRNA–miRNA network identified miRNAs like hsa-miR-129-5p, which exhibits high sensitivity and specificity in detecting patients with heart failure with reduced ejection fraction (HFrEF) (Chen et al., 2022). These networks offer valuable insights into how transcriptional and post-transcriptional mechanisms may drive the pathophysiology of AF and HF.

In summary, our findings revealed five significant genes: *CP, STEAP3, SAT1, TFRC,* and *LPCAT3.* Among them, *CP* and *STEAP3* might be used as highly correlated biomarkers of AF and HF, providing new insights into their common pathogenesis and offering therapeutic targets for patients with coexisting AF and HF. However, our study has limitations, First, the number of cases we studied was relatively small. The result based on the relatively small number of cases needs to be validated in a larger clinical sample. Secondly, we only studied the mRNA expression levels of the hub genes in serum. Further validation in cardiac tissues and *in vitro* models is required to investigate alterations of these hub genes and their underlying mechanisms during the progression of atrial fibrillation and heart failure. Nevertheless, our findings may provide novel marker genes for the prognosis and underlying mechanisms of atrial fibrillation and heart failure (AF/HF). Furthermore, this work may lay the theoretical foundation for further investigations.

## 5 Conclusion

We identified two ferroptosis genes that are highly correlated with AF and HF: *CP* and *STEAP3*. Our findings provide a theoretical basis for the clinical diagnosis and treatment of AF and HF. These results provide valuable insights into potential biomarkers for diagnosis and targets for therapeutic intervention in AF and HF.

## Data Availability

The datasets presented in this study can be found in online repositories. The names of the repository/repositories and accession number(s) can be found in the article/Supplementary Material.
