# Supplementary material for: Bioinformatics analysis and experimental validation of ferroptosis genes in heart failure and atrial fibrillation
Source: Front Genet. 2025 Jul 2;16:1541342. doi: 10.3389/fgene.2025.1541342 (PMC12263363; doi:10.3389/fgene.2025.1541342)
Supplement: Supplementary file 1 [file Table1.docx]

**Supplementary Table 1.** GEO Dataset Information list**.**

|  | GSE2240 | GSE21610 |
| --- | --- | --- |
| Platform | GPL96 | GPL570 |
| Experiment type | Expression profiling by array | Expression profiling by array |
| Species | Homo sapiens | Homo sapiens |
| Tissue | myocardium | myocardium |
| Samples in Control group | Control （5） | Control（8） |
| Samples in Disease group | AF（10） | HF（30） |
| Reference（PMID） | [15877233](https://www.ncbi.nlm.nih.gov/pubmed/15877233) | [20460602](https://www.ncbi.nlm.nih.gov/pubmed/20460602) |
