# Supplementary material for: Bioinformatics analysis and experimental validation of ferroptosis genes in heart failure and atrial fibrillation
Source: Front Genet. 2025 Jul 2;16:1541342. doi: 10.3389/fgene.2025.1541342 (PMC12263363; doi:10.3389/fgene.2025.1541342)
Supplement: Supplementary file 3 [file Table8.docx]

|  | Age (year) | Gender | Left Ventricular Ejection Fractions（%） | NT-ProBNP(pg/ml) |
| --- | --- | --- | --- | --- |
| AF 1 | 69 | Male | 61 | 307.5 |
| AF 2 | 82 | Male | 70 | 883.7 |
| AF 3 | 85 | Female | 59 | 1123 |
| AF 4 | 80 | Female | 67 | 1388 |
| AF 5 | 58 | Female | 58 | 705 |
| AF 6 | 79 | Male | 73 | 1422 |
| AF 7 | 66 | Male | 69 | 494 |
| AF 8 | 67 | Male | 63 | 714.8 |
| AF 9 | 77 | Female | 51 | 1282 |
| HF 1 | 53 | Male | 24 | 3525 |
| HF 2 | 66 | Male | 30 | 4862 |
| HF 3 | 55 | Male | 16 | 20338 |
| HF 4 | 71 | Female | 28 | 2034 |
| HF 5 | 73 | Female | 64 | 14387 |
| HF 6 | 82 | Female | 58 | 16836 |
| HF 7 | 81 | Male | 57 | 15686 |
| HF 8 | 34 | Male | 25 | 7226 |
| HF 9 | 81 | Male | 48 | 8409 |
| Control 1 | 45 | Female | 75 | 46.31 |
| Control 2 | 60 | Male | 78 | 30.5 |
| Controll 3 | 51 | Female | 71 | 41.95 |
| Control 4 | 78 | Male | 79 | 123.1 |
| Control 5 | 33 | Male | 65 | 42.3 |
| Controll 6 | 58 | Female | 64 | 91.3 |
| Control 7 | 82 | Female | 64 | 375.9 |
| Control 8 | 51 | Male | 75 | 70.98 |
| Control 9 | 65 | Male | 58 | 151.9 |

Supplementary Table8. Case information for qPCR detection of the five critical genes.AF:atrial fibrillation; HF: heart failure; NT-proBNP:**N-terminal pro-B-type natriuretic peptide**

****Inclusion Criteria:****

****Heart Failure (HF)****: Symptoms/signs of HF **and** Meeting ≥1 of the following:1.Symptoms/signs of HF **and** left ventricular ejection fraction (LVEF) <40%;2.LVEF 40–49% **with** elevated natriuretic peptides **plus** left ventricular hypertrophy (LVH) and/or left atrial enlargement (LAE) **or** diastolic dysfunction;3.LVEF ≥50% **with** elevated natriuretic peptides **plus** LVH and/or LAE **or** diastolic dysfunction.

NT-proBNP (defined as meeting ≥1 criterion):450 pg/mL (age <50 years),900 pg/mL (age 50–75 years),1800 pg/mL (age >75 years).

****Atrial Fibrillation (AF)****:Persistent AF **without** meeting the above HF criteria.

****Control**** :Individuals with **no clinically confirmed HF or AF**.
