# Supplementary material for: Bioinformatics analysis and experimental validation of ferroptosis genes in heart failure and atrial fibrillation
Source: Front Genet. 2025 Jul 2;16:1541342. doi: 10.3389/fgene.2025.1541342 (PMC12263363; doi:10.3389/fgene.2025.1541342)
Supplement: Supplementary file 5 [file Table7.docx]

## Supplementary Table 7. GSEA enrichment analysis results in HF_Dataset.

| ID | Description | setSize | enrichmentScore | NES | pvalue | p.adjust | qvalue |
| --- | --- | --- | --- | --- | --- | --- | --- |
| PID_IL12_2PATHWAY | PID_IL12_2PATHWAY | 60 | 0.58065685 | 2.11857988 | 5.7686E-06 | 0.00476682 | 0.00454204 |
| BIOCARTA_NO2IL12_PATHWAY | BIOCARTA_NO2IL12_PATHWAY | 15 | 0.77990977 | 2.06544059 | 4.6588E-05 | 0.01443657 | 0.01375583 |
| WP_REGULATION_OF_WNT_BCATENIN_SIGNALING_BY_SMALL_MOLECULE_COMPOUNDS | WP_REGULATION_OF_WNT_BCATENIN_SIGNALING_BY_SMALL_MOLECULE_COMPOUNDS | 16 | 0.72131669 | 1.93758276 | 0.00075013 | 0.07345976 | 0.06999584 |
| BIOCARTA_IL12_PATHWAY | BIOCARTA_IL12_PATHWAY | 19 | 0.68054477 | 1.90555642 | 0.00069414 | 0.07169882 | 0.06831793 |

HF: Heart failure; GSEA: Gene Set Enrichment Analysis.
