# Supplementary material for: Bioinformatics analysis and experimental validation of ferroptosis genes in heart failure and atrial fibrillation
Source: Front Genet. 2025 Jul 2;16:1541342. doi: 10.3389/fgene.2025.1541342 (PMC12263363; doi:10.3389/fgene.2025.1541342)
Supplement: Supplementary file 6 [file Table2.docx]

**Supplementary Table 2.** The qPCR primers of FRDEGs**.**

| **Genes** | **Forward (5′-3′)** | **Reverse (5′-3′)** |
| --- | --- | --- |
| CP | 5′-GGGCCATCTACCCTGATAACA-3′ | 5′-TTAAAGGTCCGATGAGTCCTGA-3′ |
| LPCAT3 | 5′-AGCCCTGATACCTTCTCTGATGG-3′ | 5′-GCATGTAGCCTGGAACTGTGTAG-3′ |
| TFRC | 5′-GGAGTGCTGGAGACTTTGGA-3′ | 5′-TATACAACAGTGGGCTGGCA-3′ |
| STEAP3 | 5′-TGCAAACTCGCTCAACTGGAG-3′ | 5′-GAAGGTGGGAGGCAGGTAGAA-3′ |
| SAT1 | 5′-ACCCGTGGATTGGCAAGTTAT-3′ | 5′-TGCAACCTGGCTTAGATTCTTC-3′ |
