# Supplementary material for: Bioinformatics analysis and experimental validation of ferroptosis genes in heart failure and atrial fibrillation
Source: Front Genet. 2025 Jul 2;16:1541342. doi: 10.3389/fgene.2025.1541342 (PMC12263363; doi:10.3389/fgene.2025.1541342)
Supplement: Supplementary file 7 [file Table5.docx]

## Supplementary Table5: GO/KEGG enrichment analysis results.

| ONTOLOGY | ID | Description | GeneRatio | BgRatio | pvalue | p.adjust | qvalue |
| --- | --- | --- | --- | --- | --- | --- | --- |
| BP | GO:0006826 | iron ion transport | 3/6 | 59/18800 | 5.8327E-07 | 0.00016681 | 6.5694E-05 |
| BP | GO:0055072 | iron ion homeostasis | 3/6 | 85/18800 | 1.7666E-06 | 0.00025262 | 9.9486E-05 |
| BP | GO:0000041 | transition metal ion transport | 3/6 | 101/18800 | 2.9749E-06 | 0.00027281 | 0.00010744 |
| BP | GO:0055076 | transition metal ion homeostasis | 3/6 | 139/18800 | 7.7831E-06 | 0.00044519 | 0.00017532 |
| BP | GO:0033572 | transferrin transport | 2/6 | 10/18800 | 3.8155E-06 | 0.00027281 | 0.00010744 |
| CC | GO:0072562 | blood microparticle | 2/6 | 147/19594 | 0.00082215 | 0.02137597 | 0.01471221 |
| CC | GO:0005771 | multivesicular body | 1/6 | 63/19594 | 0.01913964 | 0.0968253 | 0.0666409 |
| CC | GO:0005905 | clathrin-coated pit | 1/6 | 70/19594 | 0.02124729 | 0.0968253 | 0.0666409 |
| CC | GO:0030669 | clathrin-coated endocytic vesicle membrane | 1/6 | 72/19594 | 0.02184878 | 0.0968253 | 0.0666409 |
| CC | GO:0045334 | clathrin-coated endocytic vesicle | 1/6 | 91/19594 | 0.0275476 | 0.0968253 | 0.0666409 |
| MF | GO:0016722 | oxidoreductase activity, acting on metal ions | 2/6 | 17/18410 | 1.2012E-05 | 0.00052855 | 3.7934E-05 |
| MF | GO:0051087 | chaperone binding | 2/6 | 106/18410 | 0.00048523 | 0.01067515 | 0.00076616 |
| MF | GO:0016747 | acyltransferase activity, transferring groups other than amino-acyl groups | 2/6 | 218/18410 | 0.00202908 | 0.02192246 | 0.00157338 |
| MF | GO:0016746 | acyltransferase activity | 2/6 | 244/18410 | 0.00253359 | 0.02192246 | 0.00157338 |
| MF | GO:0030283 | testosterone dehydrogenase [NAD(P)] activity | 1/6 | 11/18410 | 0.00358014 | 0.02192246 | 0.00157338 |
| KEGG | hsa04216 | Ferroptosis | 5/6 | 41/8164 | 1.4841E-11 | 1.7809E-10 | 1.2498E-10 |

GO: Gene Ontology; BP: Biological Process; CC: Cellular Component; MF: molecular function; KEGG: Kyoto Encyclopedia of Genes and Genomes.
