# Supplementary material for: Bioinformatics analysis and experimental validation of ferroptosis genes in heart failure and atrial fibrillation
Source: Front Genet. 2025 Jul 2;16:1541342. doi: 10.3389/fgene.2025.1541342 (PMC12263363; doi:10.3389/fgene.2025.1541342)
Supplement: Supplementary file 8 [file Table6.docx]

## Supplementary Table 6. GSEA enrichment analysis results in AF_Dataset.

| ID | Description | setSize | enrichmentScore | NES | pvalue | p.adjust | qvalue |
| --- | --- | --- | --- | --- | --- | --- | --- |
| REACTOME_ASSEMBLY_OF_COLLAGEN_FIBRILS_AND_OTHER_MULTIMERIC_STRUCTURES | REACTOME_ASSEMBLY_OF_COLLAGEN_FIBRILS_AND_OTHER_MULTIMERIC_STRUCTURES | 54 | 0.70844754 | 2.22196879 | 1.3956E-06 | 8.37E-05 | 7.2387E-05 |
| REACTOME_DISEASES_ASSOCIATED_WITH_GLYCOSAMINOGLYCAN_METABOLISM | REACTOME_DISEASES_ASSOCIATED_WITH_GLYCOSAMINOGLYCAN_METABOLISM | 35 | 0.76117745 | 2.18296044 | 2.0842E-06 | 0.00011905 | 0.00010296 |
| WP_INFLAMMATORY_RESPONSE_PATHWAY | WP_INFLAMMATORY_RESPONSE_PATHWAY | 29 | 0.76846883 | 2.13891851 | 1.5134E-05 | 0.00069248 | 0.00059888 |
| REACTOME_MET_PROMOTES_CELL_MOTILITY | REACTOME_MET_PROMOTES_CELL_MOTILITY | 38 | 0.7232947 | 2.12002019 | 6.4642E-06 | 0.00032995 | 0.00028535 |

AF: Atrial fibrillation; GSEA: Gene Set Enrichment Analysis.
